# Structural insights into the mechanism of oxidative activation of heme-free H-NOX from *Vibrio cholerae*

**DOI:** 10.1042/BCJ20200124

**Published:** 2020-03-23

**Authors:** Roma Mukhopadhyay, Kelly N. Chacón, Jacqueline M. Jarvis, Marat R. Talipov, Erik T. Yukl

**Affiliations:** 1 Department of Chemistry and Biochemistry, New Mexico State University, Las Cruces, NM 88003, U.S.A.; 2 Department of Chemistry, Reed College, Portland, OR 97202, U.S.A.; 3 Department of Plant and Environmental Sciences, New Mexico State University, Las Cruces, NM 88003, U.S.A.

**Keywords:** disulphide bonds, redox signalling, zinc

## Abstract

Bacterial heme nitric oxide/oxygen (H-NOX) domains are nitric oxide (NO) or oxygen sensors. This activity is mediated through binding of the ligand to a heme cofactor. However, H-NOX from *Vibrio cholerae* (*Vc* H-NOX) can be easily purified in a heme-free state that is capable of reversibly responding to oxidation, suggesting a heme-independent function as a redox sensor. This occurs by oxidation of Cys residues at a zinc-binding site conserved in a subset of H-NOX homologs. Remarkably, zinc is not lost from the protein upon oxidation, although its ligation environment is significantly altered. Using a combination of computational and experimental approaches, we have characterized localized structural changes that accompany the formation of specific disulfide bonds between Cys residues upon oxidation. Furthermore, the larger-scale structural changes accompanying oxidation appear to mimic those changes observed upon NO binding to the heme-bound form. Thus, *Vc* H-NOX and its homologs may act as both redox and NO sensors by completely separate mechanisms.

## Introduction

The bacterial heme nitric oxide/oxygen (H-NOX) protein family was identified in 2003 through homology to the heme-binding domain of soluble guanylate cyclase (sGC) [1]. In mammals, sGC acts as a nitric oxide (NO) sensor, increasing levels of the secondary messenger cyclic GMP in response to NO binding to the heme iron [2]. Bacterial H-NOX proteins also bind heme and can be functionally divided into two groups based on the lifestyle of bacteria from which they originate [3]. Those found in strict anaerobes form stable Fe(II)-O_2_ species. They are fused to methyl-accepting chemotaxis proteins (MCP) and likely mediate chemotaxis in response to O_2_. Like sGC, the H-NOX proteins from facultative anaerobes do not stably bind O_2_. They modulate the activity of histidine kinases (HK) or H-NOX-associated cyclic-di-GMP processing enzymes (HaCE) in a NO-dependent manner, regulating biofilm formation [4–7], symbiont/host colonization [8] or quorum sensing [9].

One of the best-studied H-NOX/HK NO signaling systems comes from *Shewanella oneidensis*. Elegant phosphotransfer profiling experiments elucidated the signaling pathway beginning with autophosphorylation of the HK HnoK [6]. HnoK then phosphorylates the c-di-GMP phosphodiesterase HnoB, activating it to hydrolyze c-di-GMP and inhibit biofilm formation. H-NOX in the Fe(II)-NO state strongly inhibits HnoK autophosphorylation [10], resulting in an increase in cyclic-di-GMP levels and biofilm formation in response to NO. This signaling system is conserved in *Vibrio cholerae*, and autophosphorylation of *Vc* HnoK and phosphotransfer to response regulators has been confirmed [6].

As for *S. oneidensis*, holo *Vc H-NOX* in both Fe(III) and Fe(II)-NO states is a potent inhibitor of *Vc* HnoK autophosphorylation [11]. However, Fe(II) *Vc* H-NOX reacts with O_2_ [12], and the heme is sensitive to degradation in the presence of excess reductant and air [11]. Unexpectedly, the heme-free form is also a good inhibitor of HnoK autophosphorylation when reversibly activated by oxidation by hypochlorous acid (HOCl). The mechanism involves the oxidation of 4 Cys residues to form disulfide bonds. Based on homology with *S. oneidensis* H-NOX, three of these Cys co-ordinate a zinc ion along with a Gln residue in the reduced state. Remarkably, although the oxidized Cys residues are no longer able to co-ordinate zinc, metal is stoichiometrically retained by the protein after oxidation and buffer exchange. This led to the hypothesis that disulfide bond formation causes a change of zinc ligation, either or both of which may be important to mediate the conformational change that activates *Vc* H-NOX to bind and inhibit *Vc* HnoK.

Here, we have performed an analysis of H-NOX sequences to identify the conservation and diversity of putative sulfur-ligated zinc sites. We have also defined the mechanism of oxidative activation of *Vc* H-NOX, using computational methods to evaluate possible zinc coordination environments and disulfide bonding patterns in the reduced and oxidized states. These models are complemented by extended X-ray absorption fine structure (EXAFS) and mass spectrometry experiments defining the zinc ligation environment and disulfide bonding pattern, respectively. The results from computational and experimental methods are consistent and provide conclusive evidence that oxidant sensing in heme-free H-NOX proceeds via specific disulfide bond formation and reorganization of the zinc site. A comparison of the overall structural changes likely accompanying oxidation with those observed on NO binding to the heme-bound protein [13] reveal similarities that may explain how *Vc* H-NOX is able to interpret these disparate chemical signals.

## Materials and methods

### Phylogenetic analysis of H-NOX

The protein sequence of *Vc* H-NOX (UniProtKB accession number Q9KLM3) was used to perform a BLAST search of the UniProtKB database [14]. Sequences were filtered to include only those with *E*-values below 10^−20^. These sequences were submitted to the Enzyme Function Initiative — Enzyme Similarity Tool [15–17] to generate a sequence similarity network (SSN), which was further processed and visualized in Cytoscape [18]. Sequences were aligned using Clustal Omega [19].

### Conventional molecular dynamics (cMD)

The initial structure of *Vc* H-NOX [11] for MD simulation was generated by SwissProt [21] software from the X-ray crystal structure of *S. oneidensis* (So) H-NOX (PDB id: 4U99), as described in ref. [13]. MD simulations were done with the AMBER 16 package [22] using ff14SB force field [23]. The side chains of the aspartate and glutamate amino acids were in the deprotonated form in the MD simulation, and the histidine imidazoles were protonated only at the Nε atom (see additional information in Supplementary Table S1). Cysteine amino acids were in their anionic forms in the models of reduced H-NOX. For the partially and fully oxidized H-NOX models, disulfide bonds were added using the harmonic constraints on the sulfur atoms of cysteine residues. The cMD simulations have been performed using graphical processing units (GPU) [24,25]. Periodic boundary conditions were used in the MD simulations with cubic cell and a distance between any protein atom and cell boundary of at least 10 Å. Particle Mesh Ewald (PME) summation [26] was used to calculate the long-ranged electrostatic interactions with the cut-off distance of 10 Å. The protein structures were solvated by explicitly presented TIP3P water molecules [27,28]. Counter-ions (Na^+^) were added to neutralize the overall charge of the periodic cell. Time step of 4 fs was used in combination with the hydrogen mass repartitioning (HMR) [29–31] and SHAKE algorithms [32]. The SHAKE algorithm was used to restrain only the bonds containing hydrogen atoms.

Initial minimization was done with restraints on the protein atom positions for 1000 steps and then a minimization was done without restraints for 2500 steps. Then the system temperature was linearly increased using isothermal-isochoric ensemble (NVT) for 400 ps from 0 to 300 K and then equilibrated for 1 ns using isothermal-isobaric (NPT) ensemble. Finally, simulations were done in triplicates for at least 400 ns for each trajectory using the NPT ensemble. The resulting structures generated from simulations were viewed using visual molecular dynamic (VMD) software [33]. Root-mean-square difference (RMSD) analysis was performed using the least squared fitting of all the atoms of the protein backbone.

### Molecular mechanics combined with generalized Born solvation surface area approach (MM/GBSA)

The MM/GBSA method was used to compare the relative free energy between specific conformations for reduced, partially oxidized and oxidized H-NOX models by using 6000 snapshots from each MD trajectory. The water molecules and the counter-ions were removed from the produced snapshot structures. The obtained de-solvated protein structures were subjected to the potential energy evaluation using implicit solvation model within the generalized Born (GB) approximation. The calculations were performed with the pairwise GB model by Hawkins et al. [34,35], with the parameters proposed by Tsui and Case [36] and the effective salt concentration of 0.01 M.

### Umbrella sampling

Umbrella sampling [37] is a biasing sampling method used to improve the sampling of a system by monitoring the evolution of canonical MD simulation along a certain degree of freedom. Umbrella sampling for reduced H-NOX model was done by following the distance between zinc cation and the surrounding residues. A series of 27 simulation ‘windows’ were introduced for the distance between zinc and residue of interest from 1.8 to 7 Å with the step size of 0.2 Å (see Supplementary Tables S2–S8). A harmonic biasing potential of 50 kcal/(mol-Å^2^) was applied to each of the distances between the zinc and the residue of interest. Up to 17 additional sampling windows were added in case of insufficient overlap between the individual histograms for better sampling efficiency. The Newton–Raphson-based method was used for solving the weighted histogram analysis method (WHAM) equations [38].

### Fragment molecular orbital method/density functional tight binding (FMO/DFTB3)

Quantum mechanical (QM) method DFTB3 was applied using FMO code distributed in the US Gamess package [39,40]. FMO [41,42] was used to calculate energy and energy gradient faster than traditional QM methods by dividing large molecular systems into small fragments and implementing self-consistent density functional calculations of the fragment monomers and dimers. The fragments of the proteins were generated using the software Facio [43–46] by cleaving the protein backbone in between the C and C^α^ atoms in such a way that each fragment contained two neighboring amino acids with the exception of disulfide bridges whose fragments contained four amino acids. The polarized continuum model (PCM) was used to take the solvation effects into account [47]. The convergence criterion for the self-consistent charge field chosen was 10^−9 ^a.u. For the FMO/DFTB3 calculations, 108 snapshots, evenly distributed along the MD simulation course, were used. The resulting distributions of the electronic potential energies for the partially oxidized H-NOX models were plotted as probability density plots using R platform [48,49] and Jupyter notebook [50,51].

### Expression and purification of proteins

Full-length, untagged, heme-free *Vc* H-NOX was expressed from BL21 *Escherichia. coli* cells and purified by anion exchange and size exclusion chromatography as previously described [11]. Protein was stored in HEPES storage buffer (50 mM HEPES pH 8.0, 300 mM NaCl, 5% glycerol).

### Extended X-ray absorption fine structure (EXAFS)

Reduced WT *Vc* H-NOX was generated by incubating 1.3 ml of protein at 300 μM with 5 mM DTT in HEPES storage buffer for 30 min on ice and desalting into HEPES storage buffer using a 5 ml HiTrap® desalting column (GE Healthcare). The oxidized protein was generated by incubating 91.6 μM of the reduced and desalted protein with 1 mM HOCl for 1 h at room temperature followed by desalting as above. The samples were concentrated to ∼100 μl using centrifugal filtration devices (Millipore) resulting in final concentrations for reduced and oxidized samples of 1.35 mM and 0.93 mM, respectively. Ninety microliters of protein or buffer was combined with 10 μl ethylene glycol, loaded into EXAFS cuvettes and flash frozen in liquid nitrogen.

X-ray absorption data were collected at the Stanford Synchrotron Radiation Lightsource. Extended X-ray absorption fine structure (EXAFS) of Zn (9658 eV) was measured on beamlines 9–3 and 7–3 in duplicate when available using a Si 220 monochromator with crystal orientation *φ* = 0° to reduce the likelihood of known crystal glitches in the Zn energy range. Samples were measured as frozen aqueous glasses in 10% ethylene glycol at 15 K, and the X-ray absorbance was detected as Kα fluorescence using either a 100-element (beamline 9–3) or 30-element (beamline 7–3) Canberra Ge array detector. A Z-1 metal oxide filter (Cu) and Soller slit assembly was placed in front of the detector to attenuate the elastic scatter peak. Four to six scans of a buffer blank were measured at the absorption edge and subtracted from the raw data to produce a flat pre-edge and eliminate residual Cu Kβ fluorescence of the metal oxide filter. Energy calibration was achieved by placing a Zn metal foil between the second and third ionization chamber. Data reduction and background subtraction were performed using EXAFSPAK [52]. The data from each detector channel were inspected for drop outs and glitches before being included into the final average. EXAFS simulation was carried out using the program EXCURVE 9.2 as previously described [53–55].

### Trypsin digest and mass spectrometry

Fully reduced H-NOX was generated by incubating 250 μM protein with 5 mM DTT in HEPES storage buffer for 30 min at room temperature. DTT was then removed by desalting using Zeba™ spin desalting columns (Pierce). Oxidized protein was generated by incubating 150 μM reduced and desalted protein with 1.5 mM HOCl in HEPES storage buffer followed again by desalting. N-ethyl maleimide (NEM) and guanidinium-HCl were added to 4 mM and 6 M, respectively, and samples heated to 37°C for 1 h. Samples were diluted with dI water and concentrated buffer to reach a final concentration of 1 M guanidinium-HCl, 50 mM Tris pH 8.0, 20 mM CaCl_2_. Trypsin-ultra^TM^ (New England Biolabs) was added at a mass ratio of 1 : 50 and incubated at 37°C overnight. The digest was quenched by the addition of formic acid to 1% v/v. Samples were passed through a 0.2 μm filter prior to injection onto a C18 column (Sunfire 3.5 μm, 3 × 150 mm) connected to a HPLC system (Agilent). A gradient of 5–55% acetonitrile with 0.1% formic acid was applied over 40 min at 0.5 ml/min and 0.5 ml fractions were collected. Fractions with low abundance peptides were dried under a stream of nitrogen gas and reconstituted in 50 μl of 50% acetonitrile, 0.1% formic acid.

Samples were directly infused using an automated electrospray ionization (ESI) robot (Advion Nanomate) coupled to an Orbitrap Fusion mass spectrometer. An instrument method was created to automatically collect data-dependent tandem MS spectra for the peptide of interest. Positive-ion broadband ESI spectra (5 microscans) were collected from *m*/*z* 375 to 2000 at a resolving power of 120 000 at *m*/*z* 200 and AGC target of 400 000. The ions of interest, *m*/*z* 989.24 (+4 charge state), were selected for fragmentation using EThcD with the following conditions: quadrupole isolation window = 1.6, 50 ms reaction time, reagent target = 1 000 000, max reagent injection time = 200 ms, 25% supplemental activation collision energy, AGC target = 10 000, max injection time = 200 ms and ion trap detection of single microscans. MS^3^ spectra were collected from the fragmentation of the product ions from the MS^2^ spectra using the following conditions: quadrupole isolation = 2.5, MS^2^ mass isolation window = *m*/*z* 2, HCD activation at 29–29.5% collision energy, AGC target = 10 000, max injection time = 200 ms and ion trap detection of 10 microscans.

## Results

### Conservation of zinc-binding sites

**Figure 1.**
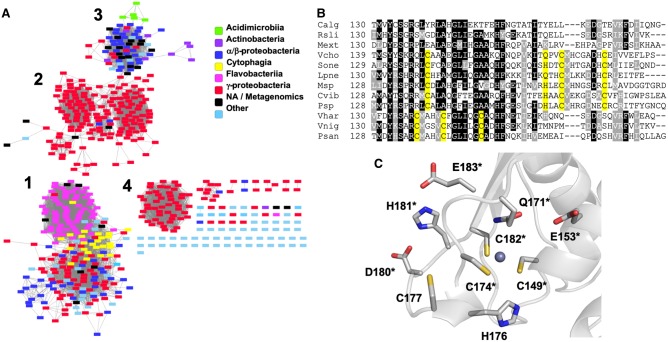
Conservation of zinc binding sites among H-NOX homologues. (**A**) Sequence similarity network including 599 sequences filtered such that only edges associated with *E*-values less than 10^−47^ are included in the network. Sequences are represented by rectangles colored according to class. (**B**) Multiple sequence alignment of three representative sequences from each group in (**A**). Putative zinc-binding residues are highlighted in yellow. Species names are abbreviated as follows: (Group 1) *Calg, Cellulophaga algiola; Rsli, Runella slithyformis; Mext, Methylobacterium extorquans*; (Group 2) *Vcho, Vibrio cholerae; Sone, Shewanella oneidensis; Lpne, Legionella pneumophila*; (Group 3) *Msp, Mycobacterium sp.; Cvib, Caulobacter vibrioides; Psp, Pseudomonas sp. DY-1*; (Group 4) *Vhar, Vibrio harveyi; Vnig, Vibrio nigripulchritudo; Psan, Photobacterium sanguinicancri*. (**C**) Zinc coordination environment in a *Vc* H-NOX homology model. Each of the residues in the figure has been mutated to Ala and those indicated by asterisks result in insoluble protein.

It was previously noted that the three zinc-binding Cys residues identified in *S. oneidensis* (Cys139, Cys164 and Cys172) were conserved among roughly half of the available *hnox* sequences from gammaproteobacteria where the fourth ligand (His161) is conserved as either Gln or His [13]. To analyze the degree of conservation of potential zinc-binding sites, a BLASTP search was performed using the *Vc* H-NOX sequence. 598 sequences were identified with a BLAST score of ≤ 10^−20^ that fall into four main groups based on a SSN (Figure 1A). The proximal His ligand to the heme (His103 in *S. oneidensis*) is absolutely conserved across all sequences. Group 1 is composed of sequences from diverse species and there is no indication of a conserved Cys-ligated zinc site (Figure 1B). Group 2 is predominantly composed of sequences from gammaproteobacteria including *S. oneidensis* and *V. cholerae*. Here the 3 Cys ligands to zinc are very highly conserved, and the fourth ligand to zinc in *S. oneidensis* (His161) is conserved as either His or Gln. Interestingly, this is also true of sequences from group 3, even though only a few sequences derive from gammaproteobacteria, suggesting that zinc coordination by H-NOX proteins is not confined to this bacterial class. Finally, group 4 is composed entirely of gammaproteobacteria from the *Vibrio* and *Photobacteria* genera. While the Cys residues from groups 2 and 3 are not conserved in group 4, a distinct pattern of 3 Cys residues is very highly conserved in this group. To date, none of these proteins have been characterized *in vitro*, so it will be of interest to determine whether these may also bind zinc or another metal through this Cys motif.

To estimate the zinc-binding environment of *Vc* H-NOX, we previously generated a homology model using the homolog from *S. oneidensis* [11], its closest structurally characterized relative. Apart from the expected apparent coordination by Cys149, Cys172, Cys182 and Gln171, a large number of potential metal-binding residues were also identified (Figure 1C). None are highly conserved. Nevertheless, they were systematically mutated to Ala in order to determine whether any may have a role in zinc coordination, particularly when the Cys ligands are oxidized. In addition to the previously described C177A mutant, only H176A was found to be expressed in soluble form in *E. coli*. H176A *Vc* H-NOX was very similar to WT in terms of zinc quantitation, Cys oxidation and reversible HnoK inhibition (Supplementary Figure S1). Thus, His176 does not appear to have a major role in zinc coordination or redox sensing. While the role of the other residues is uncertain due to insolubility of the mutants, this result does illustrate that protein stability is very sensitive to mutations of potential metal-ligand residues at the zinc-binding site.

### Computational modeling: reduced H-NOX

The homology model of the reduced *Vc* H-NOX zinc site [11], further referred to as **R1**, was used as the starting point for classical molecular dynamics (cMD) simulations. An MD simulation of this model was run in triplicate for 7.8 µs resulting in a total 23.4 µs simulation. The microsecond-scale simulations showed that **R1** was kinetically stable, as the zinc cation remained co-ordinated with Cys149, Cys174, Cys182 and Gln171 throughout the entire simulation, and the positions of backbone atoms were nearly invariant over time (RMSD < 2.7 Å).

It might seem surprising that zinc cation is co-ordinated with Gln171 rather than His176 or His181 in the **R1** model since histidine residues are more common zinc ligands than glutamine. To explore whether zinc cation can bind with nearby histidine residues, we performed cMD simulations of the formation of zinc complexes with His176 or His181 using a temporarily enabled biasing harmonic potential to bring zinc and histidine into close proximity. The subsequent simulations with removed biasing potential led to a fast dissociation of the His176/Zn bonding within the 1.3 µs simulation (**R2**) and of the His181/Zn bonding within 400 ns simulation (**R2ʹ**). This observation suggests that His/Zn complexes in *Vc* H-NOX were kinetically unstable, particularly R2ʹ, which was consequently excluded from further analysis. A subsequent comparison of the thermodynamic stability of the **R1** and **R2** models, performed using the MM/GBSA analysis, showed that model **R1** was lower in potential energy by 4.8 ± 0.4 kcal/mol than **R2**, thus suggesting that Gln171 is a thermodynamically more favorable ligand than His176 (Supplementary Figure S2).

**Figure 2.**
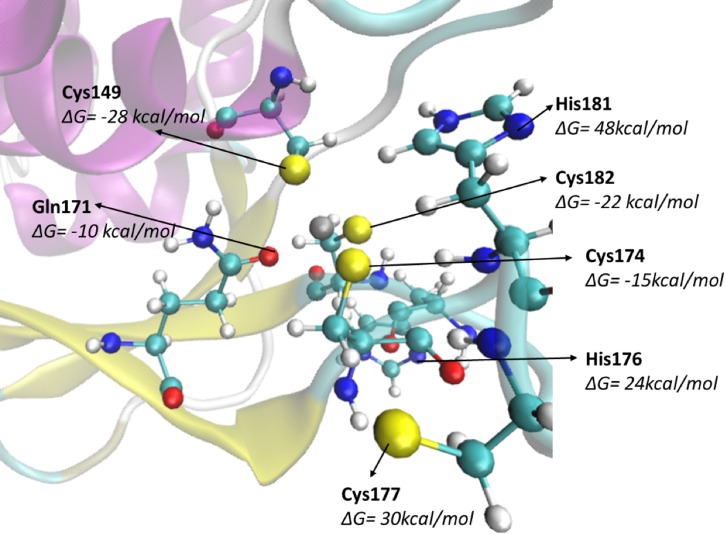
The fully reduced *Vc* H-NOX zinc binding site. (**A**) Zinc site of the thermodynamically and kinetically stable form of reduced H-NOX **R1** with the residues near zinc site and the corresponding free energy required for the residues to interact with zinc.

To solidify the results obtained from the MM/GBSA analysis on the thermodynamic stability of **R1**, a free energy profile of the formation of the bond between the zinc cation and its ligand was constructed using the umbrella sampling technique. The distance between zinc and the residue of interest was taken as the reaction coordinate to plot the potential of mean force (PMF) diagram (Supplementary Figure S3). The computed PMF plots demonstrated that zinc binds exothermically to Cys174 (Δ*G* = −15 kcal/mol), Cys149 (−28 kcal/mol), Cys182 (−22 kcal/mol) and Gln171 (−10 kcal/mol) but not with Cys177 (Δ*G* = +30 kcal/mol), H176 (+24 kcal/mol) or H181 (+48 kcal/mol). Thus, the cMD simulations, MM/GBSA results and umbrella sampling PMF plots for reduced H-NOX strongly indicated that zinc in reduced H-NOX was co-ordinated by Cys149, Cys174, Cys182 and Gln171 amino acid side chains (Figure 2).

### Computational modeling: partially oxidized H-NOX

In *Vc* H-NOX, three cysteine residues that directly interact with zinc and one additional cysteine (Cys177) in proximity to the zinc-binding site are present. Partial oxidation of *Vc* H-NOX can produce a single disulfide bond between two cysteines, however, the identity of these cysteines involved in the disulfide bonds for the partially or fully oxidized H-NOX was not known. Combination of all possible cysteine residues to form a single disulfide bond generated six different models of partially oxidized H-NOX (**P1**, **P1ʹ**, **P2**, **P2ʹ**, **P3** and **P3ʹ**) (Table 1). The MD simulations showed that all of these models were kinetically stable for the entire simulation range of 1.2 μs (RMSD < 3.4 Å). The following MM/GBSA analysis showed that model **P1ʹ** was the most stable one while models **P3** and **P3ʹ** could be excluded from further consideration because of the obvious energetic penalty (Table 1).

**Table 1.** **Tabulated relative free energy (ΔΔ*G* = Δ*G*_calc _− Δ*G*_ref_) for various partially oxidized H-NOX models as computed by MM/GBSA. Δ*G*_ref_ = **Δ*G***_calc_(P1ʹ)**

Model	Disulfide bridge	ΔΔ*G* (kcal/mol)	Std. error
**P1**	Cys149–Cys177	26.5	1.2
**P1ʹ**	Cys174–Cys182	0.0 (reference)	—
**P2**	Cys149–Cys182	37.4	0.1
**P2ʹ**	Cys174–Cys177	8.7	0.1
**P3**	Cys149–Cys174	117.8	0.2
**P3ʹ**	Cys177–Cys182	134.1	0.2

**Figure 3.**
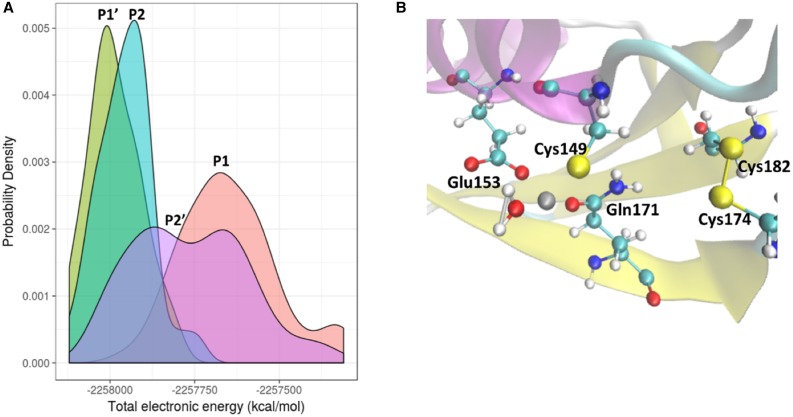
The partially oxidized *Vc* H-NOX zinc binding site. (**A**) Probability density distribution from FMO/DFTB3 results show the bond energetics in different partially oxidized models. (**B**) Zinc site in partially oxidized H-NOX model **P1ʹ**.

The MM/GBSA results, presented above, include only coulombic and van der Waals terms and neglect any contributions from the zinc-based orbital interactions. To take the orbital interactions into account, we used a QM approach FMO/DFTB3, as described in the computational methods. The distributions of the FMO/DFTB3 potential energies of the protein conformations during the MD simulation course confirmed that the Cys174/182 (**P1ʹ**) bonding interaction was the most stable one while models **P2** and **P2ʹ** were comparable in energy and at least 57 kcal/mol less stable than **P1ʹ** (Figure 3). This result suggests that the formation of the bond between Cys174 and Cys182 (that is, **R1 **→ **P1ʹ** transformation) represents the first oxidation event for reduced *Vc* H-NOX.

### Computational modeling: fully oxidized H-NOX

Three different models of fully oxidized H-NOX could be combinatorially generated, that is model **O1** (Cys174/182 and Cys149/177 bonds), **O2** (Cys149/182 and Cys174/177 bonds) and **O3** (Cys149/174 and Cys177/182 bonds). The models **O1** and **O2** were subjected to a 1.2 μs simulations and were found to be kinetically stable (RMSD < 3.7 Å) while **O3** was discarded from further consideration as it would require a formation of highly energetically unfavorable Cys149/174 (**P3**) and Cys177/182 (**P3ʹ**) interactions. The subsequent MM/GBSA analysis demonstrated that the model **O1** was energetically more favorable over **O2** by 16.6 ± 0.1 kcal/mol (Supplementary Figure S4). Thus, the computational modeling suggests that oxidation of *Vc* H-NOX leads to **P1ʹ** (Cys174/182 bond) and, subsequently, **O1** (Cys174/182 and Cys149/177 bonds).

Our previous study indicated that fully oxidized H-NOX still retains the zinc cation [11]. However, its precise location and coordination environment in the oxidized state remains unknown. In the model **O1**, Zn was originally placed at the same position where it was in the reduced form of H-NOX. The cMD simulations showed that it resides in a loosely defined pocket containing the side chain of Glu153 and backbone carbonyls of Ile184, Thr170, Val185 and Ile169. To identify whether there existed other binding regions for the zinc cation, the electrostatic potential energy surface of the oxidized H-NOX model was analyzed using Chimera [56] software (Supplementary Figure S5). Based on this analysis, we generated a series of new models of oxidized H-NOX in which zinc was placed in the regions with prominent electron-deficient character. This resulted in 11 different models, which were subjected to the cMD simulations followed by MM/GBSA calculations. However, no well-defined zinc-binding pocket could be identified despite multiple attempts. Similar simulations, conducted for the **O2** model, also showed a large variation of the zinc cation position. At the same time, the generated models were of higher energies as compared with **O1** thus solidifying the conclusion that **O1** indeed was more stable than **O2**.

### Zinc EXAFS of reduced and oxidized Vc H-NOX

**Figure 4.**
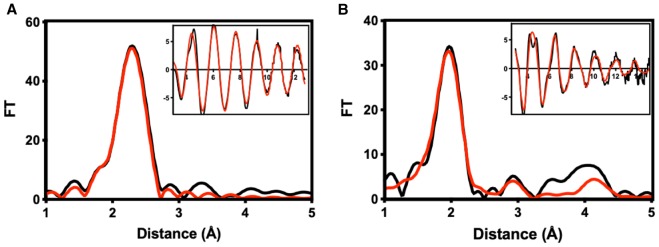
EXAFS spectra of fully reduced and fully oxidized *Vc* H-NOX. Zn K EXAFS and Fourier transforms of reduced (**A**) and oxidized (**B**) H-NOX. Black traces represent experimental data and red traces are simulations. Parameters used to generate the simulated spectra are listed in Table 2.

Zn K-edge EXAFS was employed to experimentally define the coordination environment of zinc in reduced and oxidized H-NOX. The Fourier transform (FT) of the reduced form (Figure 4A) shows a strong peak centered ∼2.314 Å consistent with sulfur ligation, with a slight shoulder at 2.014 Å due to oxygen or nitrogen ligation. The simulated EXAFS of the reduced form (Figure 4A inset) fit remarkably well to the data and indicates 3 S and 1 O/N ligands (Table 2), consistent with model **R1** (Figure 2) of H-NOX where Cys149, Cys174, Cys177 and Gln171 co-ordinate the zinc ion. Of note are the low Debye–Waller (DW) values for all four ligands, indicating that these residues do not exhibit notable flux upon binding of the metal ion. In contrast, the EXAFS of oxidized H-NOX (Figure 4B) was significantly different and exhibited weaker scattering atoms at shorter distances, which is consistent with a change from a primarily sulfur ligation sphere to one dominated by O/N ligands. Indeed, EXAFS simulations indicate a complete loss of sulfur ligation in favor of a new tetra or penta-co-ordinate environment. We are able to best fit to an environment composed of 4 O/N ligands at 1.955 Å with a fifth multiple scattering contribution at the same distance that was best modeled by a histidine. As the DW factor for the O/N ligands were unusually high for this type of scatterer (0.015 Å^2^), it is likely that the Zn associates flexibly with those atoms, which might be expected in order for the movement to occur from the reduced to the oxidized site. This flexibility may also indicate a more biologically typical tetraco-ordinate environment, which includes a stably associated histidine. While this coordination does not precisely match any of the zinc sites in modeled oxidized structures, the EXAFS data conclusively demonstrates a marked change in zinc coordination from primarily sulfur to primarily O/N upon oxidation. Desalting the oxidized sample into the fresh buffer prior to EXAFS data collection allows us to rule out contributions of any aquo- or buffer-zinc complex that might form as a result of dissociation from the protein, which was not observed to any significant degree in our previous study [11].

**Table 2.** Fit parameters for Zn EXAFS simulations

	Fl	No	*R* (Å)	DW (Å^2^)	No	*R* (Å)	DW (Å^2^)	No	*R* (Å)	DW (Å^2^)	*E*_0_
Zn K		Zn-S			Zn-N/O			Zn-N(His)			
Red	0.27	3	2.314	0.004	1	2.014	0.002				−11.97
Ox	0.47				4	1.955	0.015	1	1.995	0.003	−11.37

### Mass spectrometry identification of the disulfide bonding pattern in oxidized Vc H-NOX

Our previous work combined with the above results demonstrates that oxidation results in complete loss of Cys ligation to the zinc and the formation of two disulfide bonds. Since 3 out of 4 Cys residues are housed on the same tryptic peptide, a tandem mass spectrometry (MS^3^) protocol similar to that previously published [57] was developed to determine the disulfide bonding pattern. The protocol employs electron transfer dissociation with supplemental high collision energy activation (EThcD; MS^2^) and high collision-induced dissociation (HCD; MS^3^) to differentiate between intra- and inter-peptide disulfide bonds. ETD and the related electron capture dissociation (ECD) preferentially fragment disulfide bonds over peptide bonds [58–60]. Subsequent analysis of the resulting peptides by HCD fragmentation at peptide bonds by MS^3^ allows for their identification. Furthermore, since disulfide bonds are not typically fragmented by CID [61], no fragmentation is observed at residues within an intrapeptide disulfide. Similar approaches have been used to assign complex disulfide bonding patterns in antibodies [57,62–64]. Our approach depends on the ability to fragment the inter-peptide disulfide bond by EThcD and select for the resultant fragment with the intrapeptide bond still intact for HCD-MS^3^. A window of residues between Cys residues will not fragment in the MS^3^ step and identify the cysteines involved in this bond.

**Figure 5.**
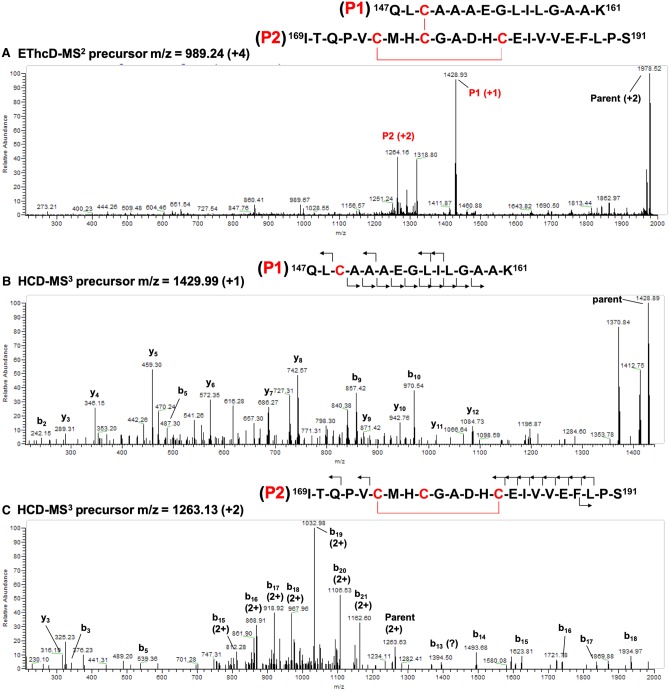
Mass spectra of the disulfide cross-linked peptide of oxidized H-NOX. (**A**) EThcD-MS2 spectrum of *m*/*z* 989.24 (+4). (**B**) HCD-MS3 spectra of the P1 peptide with *m*/*z* 1429.99 (+1) and (**C**) the P2 peptide with *m*/*z* 1263.13 (+2). The peptide sequences with the observed fragment ions are shown above each spectrum with Cys residues and disulfide bonds indicated in red.

In oxidized WT H-NOX, the cross-linked +4 parent ion with *m*/*z* = 989.24 was positively identified from offline HPLC fractions. Notably, this ion was not identified in reduced H-NOX mass spectra nor was its counterpart observed in oxidized C177A H-NOX. ETD fragmentation yielded two new peptides with masses consistent with Ile169-Ser191 (P1) and Gln147-Lys161 (P2) (Figure 5A), indicating that ETD ruptured the inter-peptide disulfide bond. These assignments were confirmed by HCD fragmentation (Figure 5B,C, Supplementary Tables S9,S10). Furthermore, the HCD fragmentation pattern of Ile169-Ser191 is consistent with an intra-disulfide bond between Cys174 and Cys182 as virtually no fragments are observed between these two residues. A possible b_13_ fragment was identified, which would indicate cleavage between Cys182 and His181. However, the *m*/*z* is 1 unit larger than expected for this fragment, even assuming all Cys residues are protonated (Supplementary Table S10). It is possible that this is due to a small amount of P2 peptide with both disulfide bonds severed during EThcD fragmentation. Alternatively, it may be due to neutral loss from an unidentified c, z, y, or b-ion. In any case, no other y- or b-ions for fragments between Cys174 and Cys182 could be identified. Taken together, the mass spectrometry data strongly suggests that the disulfide bonding pattern in oxidized H-NOX is between Cys174–Cys182 and Cys177–Cys149 as indicated in Figure 5A and predicted computationally (Supplementary Figure S4).

## Discussion

Structural studies on the H-NOX family have made significant progress toward understanding the mechanisms of signal transduction [65]. Briefly, NO binding to the heme iron displaces the proximal His ligand, relieving heme distortion imposed by steric constraints with a conserved, proximal Pro residue. The displaced proximal His rotates out of the pocket ∼90° along with helix αF. These perturbations lead to a small rigid-body displacement and rotation of the distal subdomain relative to the proximal subdomain. The rotation occurs about a hinge composed of two conserved Gly residues (G81 and G154 in *Vc* H-NOX). While the initial events differ between NO and O_2_ sensing H-NOX proteins, Pro-heme interactions and subdomain realignment appear to be conserved, at least between the NO-sensing H-NOX from *S. oneidensis* [13,66] and the O_2_-sensing H-NOX from *Caldanaerobacter subterraneus* [67–70]. Hydrogen-deuterium exchange mass spectrometry (HDX-MS) experiments indicate that *So* H-NOX [71] and *Vc* H-NOX [72] interact with their respective histidine kinases (HnoK) through the distal subdomain helices αA-αC and proximal subdomain helix αF. This supports the idea that reorientation of subdomains modulates the interaction between H-NOX and HnoK, mediating autokinase inhibition in the presence of the appropriate signal.

**Figure 6.**
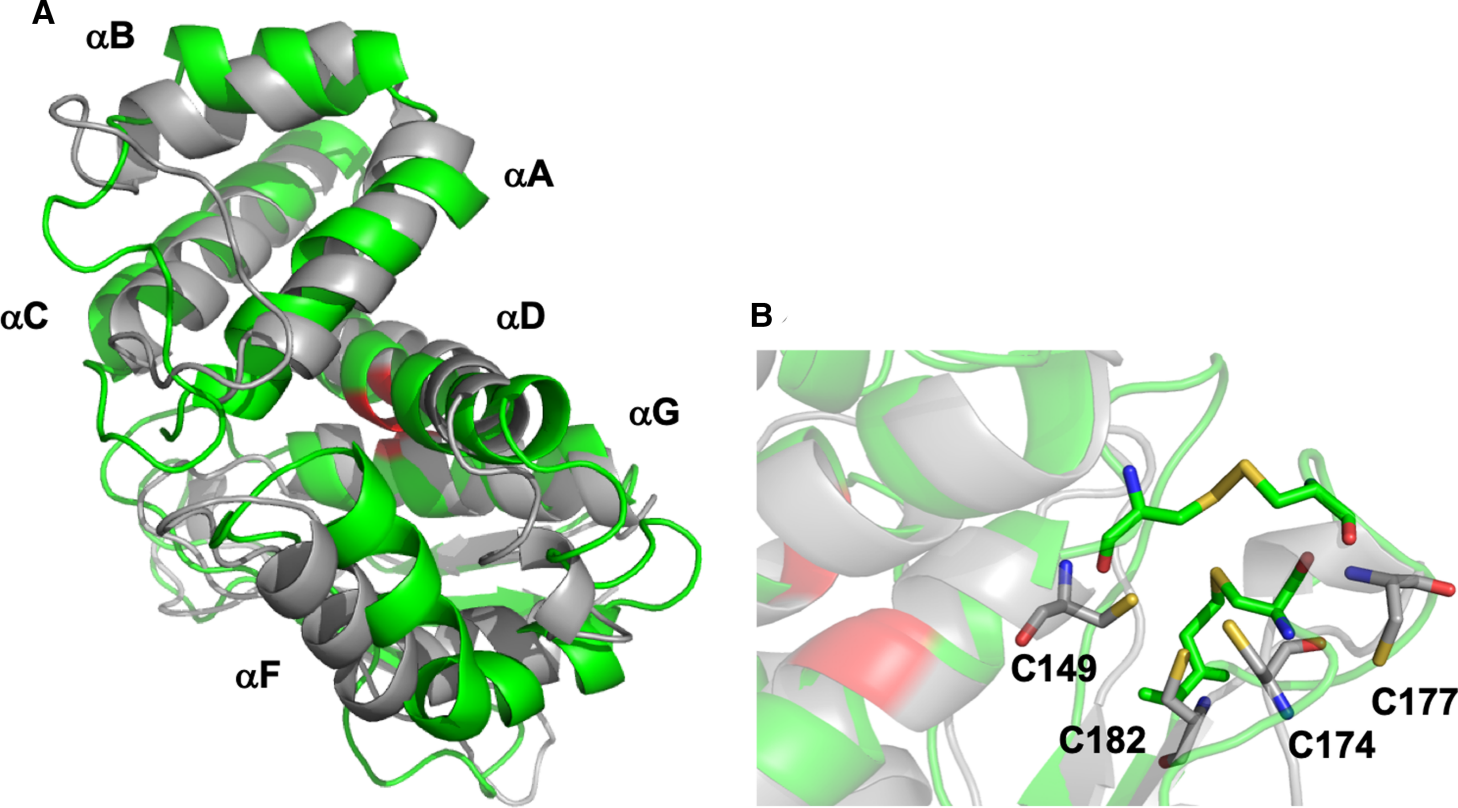
Models of heme-free reduced (gray) and oxidized (green) *Vc* H-NOX. (**A**) Overall structures showing positions of important alpha helices. (**B**) Reduced zinc-binding site. Zinc has been omitted for clarity. Cys residues are shown as sticks colored according to element. Gly81 and Gly154 at the glycine switch are colored red.

Intriguingly, a comparison of the lowest energy models of reduced and oxidized heme-free apo H-NOX reveal significant changes at αA-αC, αF and the glycine hinge region (Figure 6A). In this case, these motions are likely mediated by the impact of disulfide bond formation on the nearby glycine hinge (Figure 6B). In particular, C149 must move ∼5 Å to form a disulfide bond with C177. The position of C149 at the N-terminal end of αG only 5 residues from G154 suggests that this motion will be communicated to the glycine hinge, promoting reorientation of the subdomains. Based on these models and our previous biochemical data, it seems likely that disulfide bond formation in heme-free H-NOX may mimic motions accompanying NO binding to the heme iron, resulting in a conformation of H-NOX capable of inhibiting HnoK autophosphorylation.

The reversible formation of disulfide bonds at Cys-ligated zinc sites is proposed to mediate redox sensing in many proteins [73]. To our knowledge, H-NOX is unique in that the zinc remains bound to the protein in a new coordination environment, although the precise binding site in the oxidized protein and its importance in HnoK inhibition remain unclear. Given the EXAFS data (Figure 4) and the results of mutagenesis near the zinc site, it is tempting to speculate that nearby His and Asp/Glu residues co-ordinate zinc in the oxidized state and are essential for stability. Unfortunately, a computational model corroborating this assertion could not be generated. This may be due to several reasons, including that (1) the initial homology H-NOX model could be not sufficiently accurate for the MD simulations and (2) the binding site in the oxidized protein is formed by slow conformational changes that could not be tracked with the microsecond-scale MD simulations.

Similarly, whether *Vc* H-NOX mediates biofilm formation in response to oxidative stress *in vivo* through this mechanism is uncertain. However, several studies have shown an increase of reactive oxygen species (ROS) in cholera patients relative to uninfected persons [74–76], and the expression of biofilm genes is important for various models of intestinal colonization [77–80]. Furthermore, biofilm-like aggregates of semi-dormant but infectious cells have been found in cholera patient stools and in the aquatic environment [81,82]. Thus, there does seem to be a relationship between biofilm formation and oxidative stress in *V. cholerae* similar to that confirmed in *Campylobacter jejuni* [83], another gut pathogen. It is possible that *Vc* H-NOX may play a role in redox sensing leading to biofilm formation, with potentially important implications on transmission and pathogenesis of cholera. Further studies will be required to evaluate this possibility.

In conclusion, we have confirmed and refined the mechanism of redox sensing by heme-free *Vc* H-NOX using experimental and computational tools. The results delineate the disulfide bonding pattern in the oxidized protein and confirm a dramatic change in zinc coordination environment. The overall conformational changes upon oxidation mimic those observed upon NO binding to the heme-bound protein and explain why cysteine oxidation results in HnoK inhibition. Given that the zinc-ligating Cys residues are conserved in several H-NOX homologs, it will be interesting to determine whether this mechanism is active in other members of the H-NOX family. Further experiments assessing this as well as the *in vivo* function of heme-independent redox signaling in H-NOX proteins promise to expand our understanding of the diversity of functions in this important sensor family.

## Abbreviations

cMD: classical molecular dynamics
DW: Debye–Waller
ESI: electrospray ionization
EXAFS: extended X-ray absorption fine structure
FMO/DFTB3: fragment molecular orbital method/density functional tight binding
GB: generalized born
HCD: high collision-induced dissociation
HK: histidine kinases
H-NOX: heme nitric oxide/oxygen
HOCl: hypochlorous acid
MM/GBSA: molecular mechanics combined with generalized Born solvation surface area approach
MS^3^: mass spectrometry
NO: nitric oxide
PMF: potential of mean force
QM: quantum mechanical
RMSD: root-mean-square difference
sGC: soluble guanylate cyclase
SSN: sequence similarity network

## References

[1] Iyer L.M., Anantharaman V. and Aravind L. (2003) Ancient conserved domains shared by animal soluble guanylyl cyclases and bacterial signaling proteins. BMC Genomics 4, 5 10.1186/1471-2164-4-5 12590654 PMC149354

[2] Lucas K.A., Pitari G.M., Kazerounian S., Ruiz-Stewart I., Park J., Schulz S. et al. (2000) Guanylyl cyclases and signaling by cyclic GMP. Pharmacol. Rev. 52, 375–414 PMID: 10977868

[3] Boon E.M. and Marletta M.A. (2005) Ligand specificity of H-NOX domains: from sGC to bacterial NO sensors. J. Inorg. Biochem. 99, 892–902 10.1016/j.jinorgbio.2004.12.016 15811506

[4] Carlson H.K., Vance R.E. and Marletta M.A. (2010) H-NOX regulation of c-di-GMP metabolism and biofilm formation in *Legionella pneumophila*. Mol. Microbiol. 77, 930–942 10.1111/j.1365-2958.2010.07259.x 20572940 PMC2952683

[5] Liu N., Xu Y., Hossain S., Huang N., Coursolle D., Gralnick J.A. et al. (2012) Nitric oxide regulation of cyclic di-GMP synthesis and hydrolysis in *Shewanella woodyi*. Biochemistry 51, 2087–2099 10.1021/bi201753f 22360279

[6] Plate L. and Marletta M.A. (2012) Nitric oxide modulates bacterial biofilm formation through a multicomponent cyclic-di-GMP signaling network. Mol. Cell 46, 449–460 10.1016/j.molcel.2012.03.023 22542454 PMC3361614

[7] Kumar S. and Spiro S. (2017) Environmental and genetic determinants of biofilm formation in *Paracoccus denitrificans*. mSphere 2, e00350-17 10.1128/mSphereDirect.00350-17 28904996 PMC5588039

[8] Wang Y., Dufour Y.S., Carlson H.K., Donohue T.J., Marletta M.A. and Ruby E.G. (2010) H-NOX-mediated nitric oxide sensing modulates symbiotic colonization by *Vibrio fischeri*. Proc. Natl. Acad. Sci. U.S.A. 107, 8375–8380 10.1073/pnas.1003571107 20404170 PMC2889544

[9] Henares B.M., Higgins K.E. and Boon E.M. (2012) Discovery of a nitric oxide responsive quorum sensing circuit in *Vibrio harveyi*. ACS Chem. Biol. 7, 1331–1336 10.1021/cb300215t 22606970

[10] Price M.S., Chao L.Y. and Marletta M.A. (2007) *Shewanella oneidensis* MR-1 H-NOX regulation of a histidine kinase by nitric oxide. Biochemistry 46, 13677–13683 10.1021/bi7019035 17988156 PMC2531215

[11] Mukhopadyay R., Sudasinghe N., Schaub T. and Yukl E.T. (2016) Heme-independent redox sensing by the heme-nitric oxide/oxygen-binding protein (H-NOX) from *Vibrio cholerae*. J. Biol. Chem. 291, 17547–17556 10.1074/jbc.M116.733337 27358409 PMC5016152

[12] Wu G., Liu W., Berka V. and Tsai A.L. (2013) The selectivity of *Vibrio cholerae* H-NOX for gaseous ligands follows the “Sliding Scale Rule” hypothesis. Ligand interactions with both ferrous and ferric Vc H-NOX. Biochemistry 52, 9432–9446 10.1021/bi401408x 24351060 PMC3999706

[13] Herzik M.A. Jr, Jonnalagadda R., Kuriyan J. and Marletta M.A. (2014) Structural insights into the role of iron-histidine bond cleavage in nitric oxide-induced activation of H-NOX gas sensor proteins. Proc. Natl. Acad. Sci. U.S.A. 111, E4156–E4164 10.1073/pnas.1416936111 25253889 PMC4210026

[14] Consortium U. (2019) Uniprot: a worldwide hub of protein knowledge. Nucleic Acids Res. 47, D506–D515 10.1093/nar/gky1049 30395287 PMC6323992

[15] Gerlt J.A., Bouvier J.T., Davidson D.B., Imker H.J., Sadkhin B., Slater D.R. et al. (2015) Enzyme Function Initiative-Enzyme Similarity Tool (EFI-EST): a web tool for generating protein sequence similarity networks. Biochim. Biophys. Acta 1854, 1019–1037 10.1016/j.bbapap.2015.04.015 25900361 PMC4457552

[16] Gerlt J.A. (2017) Genomic enzymology: web tools for leveraging protein family sequence-function space and genome context to discover novel functions. Biochemistry 56, 4293–4308 10.1021/acs.biochem.7b00614 28826221 PMC5569362

[17] Zallot R., Oberg N.O. and Gerlt J.A. (2018) ‘Democratized’ genomic enzymology web tools for functional assignment. Curr. Opin. Chem. Biol. 47, 77–85 10.1016/j.cbpa.2018.09.009 30268904 PMC6289791

[18] Shannon P., Markiel A., Ozier O., Baliga N.S., Wang J.T., Ramage D. et al. (2003) Cytoscape: a software environment for integrated models of biomolecular interaction networks. Genome. Res. 13, 2498–2504 10.1101/gr.1239303 14597658 PMC403769

[19] Sievers F., Wilm A., Dineen D., Gibson T.J., Karplus K., Li W. et al. (2011) Fast, scalable generation of high-quality protein multiple sequence alignments using Clustal Omega. Mol. Syst. Biol. 7, 539 10.1038/msb.2011.75 21988835 PMC3261699

[20] Waterhouse A.M., Procter J.B., Martin D.M., Clamp M. and Barton G.J. (2009) Jalview Version 2–a multiple sequence alignment editor and analysis workbench. Bioinformatics 25, 1189–1191 10.1093/bioinformatics/btp033 19151095 PMC2672624

[21] Arnold K., Bordoli L., Kopp J. and Schwede T. (2006) The SWISS-MODEL workspace: a web-based environment for protein structure homology modelling. Bioinformatics 22, 195–201 10.1093/bioinformatics/bti770 16301204

[22] Case D.A., Cheatham T.E. III, Darden T., Gohlke H., Luo R., Merz, Jr K.M. et al. (2005) The Amber biomolecular simulation programs. J. Comput. Chem. 26, 1668–1688 10.1002/jcc.20290 16200636 PMC1989667

[23] Maier J.A., Martinez C., Kasavajhala K., Wickstrom L., Hauser K.E. and Simmerling C. (2015) ff14SB: improving the accuracy of protein side chain and backbone parameters from ff99SB. J. Chem. Theory Comput. 11, 3696–3713 10.1021/acs.jctc.5b00255 26574453 PMC4821407

[24] Götz A.W., Williamson M.J., Xu D., Poole D., Le Grand S. and Walker R.C. (2012) Routine microsecond molecular dynamics simulations with AMBER on GPUs. 1. Generalized born. J. Chem. Theory Comput. 8, 1542–1555 10.1021/ct200909j 22582031 PMC3348677

[25] Salomon-Ferrer R., Götz A.W., Poole D., Le Grand S. and Walker R.C. (2013) Routine microsecond molecular dynamics simulations with AMBER on GPUs. 2. Explicit solvent particle mesh Ewald. J. Chem. Theory Comput. 9, 3878–3888 10.1021/ct400314y 26592383

[26] Essmann U., Perera L., Berkowitz M.L., Darden T., Lee H. and Pedersen L.G. (1995) A smooth particle mesh Ewald method. J. Chem. Phys. 103, 8577–8593 10.1063/1.470117

[27] Jorgensen W.L., Chandrasekhar J., Madura J.D., Impey R.W. and Klein M.L. (1983) Comparison of simple potential functions for simulating liquid water. J. Chem. Phys. 79, 926–935 10.1063/1.445869

[28] Larsson P., Hess B. and Lindahl E. (2011) Algorithm improvements for molecular dynamics simulations. Wiley Interdiscip. Rev. Comput. Mol. Sci. 1, 93–108 10.1002/wcms.3

[29] Feenstra K.A., Hess B. and Berendsen H.J.C. (1999) Improving efficiency of large time-scale molecular dynamics simulations of hydrogen-rich systems. J. Comput. Chem. 20, 786–798 10.1002/(SICI)1096-987X(199906)20:8<786::AID-JCC5>3.0.CO;2-B 35619462

[30] Harvey M.J., Giupponi G. and Fabritiis G.D. (2009) ACEMD: Accelerating biomolecular dynamics in the microsecond time scale. J. Chem. Theory Comput. 5, 1632–1639 10.1021/ct9000685 26609855

[31] Hopkins C.W., Le Grand S., Walker R.C. and Roitberg A.E. (2015) Long-time-step molecular dynamics through hydrogen mass repartitioning. J. Chem. Theory Comput. 11, 1864–1874 10.1021/ct5010406 26574392

[32] Ryckaert J.-P., Ciccotti G. and Berendsen H.J.C. (1977) Numerical integration of the cartesian equations of motion of a system with constraints: molecular dynamics of n-alkanes. J. Comput. Phys. 23, 327–341 10.1016/0021-9991(77)90098-5

[33] Humphrey W., Dalke A. and Schulten K. (1996) VMD: visual molecular dynamics. J. Mol. Graph. 14, 33–38 10.1016/0263-7855(96)00018-5 8744570

[34] Hawkins G.D., Cramer C.J. and Truhlar D.G. (1996) Parametrized models of aqueous free energies of solvation based on pairwise descreening of solute atomic charges from a dielectric medium. J. Phys. Chem. 100, 19824–19839 10.1021/jp961710n

[35] Hawkins G.D., Cramer C.J. and Truhlar D.G. (1995) Pairwise solute descreening of solute charges from a dielectric medium. Chem. Phys. Lett. 246, 122–129 10.1016/0009-2614(95)01082-K

[36] Tsui V. and Case D.A. (2000) Theory and applications of the generalized Born solvation model in macromolecular simulations. Biopolymers 56, 275–291 10.1002/1097-0282(2000)56:4<275::AID-BIP10024>3.0.CO;2-E 11754341

[37] Torrie G.M. and Valleau J.P. (1977) Nonphysical sampling distributions in Monte Carlo free-energy estimation: Umbrella sampling. J. Comput. Phys. 23, 187–199 10.1016/0021-9991(77)90121-8

[38] Kumar S., Rosenberg J.M., Bouzida D., Swendsen R.H. and Kollman P.A. (1992) THE weighted histogram analysis method for free-energy calculations on biomolecules. I. The method. J. Comput. Chem. 13, 1011–1021 10.1002/jcc.540130812

[39] Dykstra C., Frenking G., Kim K. and Scuseria G. (2011) Theory and Applications of Computational Chemistry, Elsevier

[40] Schmidt M.W., Baldridge K.K., Boatz J.A., Elbert S.T., Gordon M.S., Jensen J.H. et al. (1993) General atomic and molecular electronic structure system. J. Comput. Chem. 14, 1347–1363 10.1002/jcc.540141112

[41] Fedorov D.G., and Kitaura K (2006) Theoretical development of the fragment molecular orbital (FMO) method In Modern Methods for Theoretical Physical Chemistry of Biopolymers, pp. 3–38, Elsevier

[42] Fedorov D.G. and Kitaura K. (2007) Pair interaction energy decomposition analysis. J. Comput. Chem. 28, 222–237 10.1002/jcc.20496 17109433

[43] Inadomi Y., Nakano T., Kitaura K. and Nagashima U. (2002) Definition of molecular orbitals in fragment molecular orbital method. Chem. Phys. Lett. 364, 139–143 10.1016/S0009-2614(02)01291-5

[44] Kitaura K., Ikeo E., Asada T., Nakano T. and Uebayasi M. (1999) Fragment molecular orbital method: an approximate computational method for large molecules. Chem. Phys. Lett. 313, 701–706 10.1016/S0009-2614(99)00874-X

[45] Ishimoto T., Tokiwa H., Teramae H. and Nagashima U. (2005) Theoretical study of intramolecular interaction energies during dynamics simulations of oligopeptides by the fragment molecular orbital-Hamiltonian algorithm method. J. Chem. Phys. 122, 094905 10.1063/1.1857481 15836179

[46] Suenaga M. (2009) Facio Home Page, http://zzzfelis.sakura.ne.jp/index.html

[47] Fedorov D.G., Kitaura K., Li H., Jensen J.H. and Gordon M.S. (2006) The polarizable continuum model (PCM) interfaced with the fragment molecular orbital method (FMO). J. Comput. Chem. 27, 976–985 10.1002/jcc.20406 16604514

[48] Gandrud C. (2016) Reproducible Research with R and R Studio, Chapman and Hall/CRC

[49] Team RC. (2013) R: a Language and Environment for Statistical Computing, R Foundation for Statistical Computing, Vienna

[50] Kluyver T., Ragan-Kelley B., Pérez F., Granger B.E., Bussonnier M. Frederic J. et al. (eds) (2016) Jupyter Notebooks-a publishing format for reproducible computational workflows. Computer Science, Published in ELPUB.

[51] Ragan-Kelley M., Perez F., Granger B., Kluyver T., Ivanov P. Frederic J., et al. (eds) (2014) The Jupyter/IPython architecture: a unified view of computational research, from interactive exploration to communication and publication. AGU Fall Meeting Abstracts, Computer Science

[52] George G.N. (1995) EXAFSPAK. A suite of computer programs for analysis of X-ray absorption spectra. Stanford Synchrotron Radiation Laboratory, Menlo Park, USA

[53] Binsted N. and Hasnain S.S. (1996) State-of-the-art analysis of whole X-ray absorption spectra. J. Synchrotron. Radiat. 3(Pt 4), 185–196 10.1107/S0909049596005651 16702677

[54] Gurman S.J., Binsted N. and Ross I. (1984) A rapid, exact curved-wave theory for EXAFS calculations. J. Phys. C Solid State Phys. 17, 143–151 10.1088/0022-3719/17/1/019

[55] Gurman S.J., Binsted N. and Ross I. (1986) A rapid, exact, curved-wave theory for EXAFS calculations. II. The multiple-scattering contributions. J. Phys. C Solid State Phys. 19, 1845–1861 10.1088/0022-3719/19/11/021

[56] Pettersen E.F., Goddard T.D., Huang C.C., Couch G.S., Greenblatt D.M., Meng E.C. et al. (2004) UCSF chimera—a visualization system for exploratory research and analysis. J. Comput. Chem. 25, 1605–1612 10.1002/jcc.20084 15264254

[57] Wu S.L., Jiang H., Lu Q., Dai S., Hancock W.S. and Karger B.L. (2009) Mass spectrometric determination of disulfide linkages in recombinant therapeutic proteins using online LC–MS with electron-transfer dissociation. Anal. Chem. 81, 112–122 10.1021/ac801560k 19117448 PMC2645030

[58] Mikesh L.M., Ueberheide B., Chi A., Coon J.J., Syka J.E., Shabanowitz J. et al. (2006) The utility of ETD mass spectrometry in proteomic analysis. Biochim. Biophys. Acta 1764, 1811–1822 10.1016/j.bbapap.2006.10.003 17118725 PMC1853258

[59] Clark D.F., Go E.P. and Desaire H. (2013) Simple approach to assign disulfide connectivity using extracted ion chromatograms of electron transfer dissociation spectra. Anal. Chem. 85, 1192–1199 10.1021/ac303124w 23210856 PMC3607449

[60] McLafferty F.W., Horn D.M., Breuker K., Ge Y., Lewis M.A., Cerda B. et al. (2001) Electron capture dissociation of gaseous multiply charged ions by Fourier-transform ion cyclotron resonance. J Am. Soc. Mass Spectrom. 12, 245–249 10.1016/S1044-0305(00)00223-3 11281599

[61] Loo J.A., Edmonds C.G., Udseth H.R. and Smith R.D. (1990) Effect of reducing disulfide-containing proteins on electrospray ionization mass spectra. Anal. Chem. 62, 693–698 10.1021/ac00206a009 2327585

[62] Wang Y., Lu Q., Wu S.L., Karger B.L. and Hancock W.S. (2011) Characterization and comparison of disulfide linkages and scrambling patterns in therapeutic monoclonal antibodies: using LC–MS with electron transfer dissociation. Anal. Chem. 83, 3133–3140 10.1021/ac200128d 21428412 PMC3082428

[63] Cole S.R., Ma X., Zhang X. and Xia Y. (2012) Electron transfer dissociation (ETD) of peptides containing intrachain disulfide bonds. J. Am. Soc. Mass Spectrom. 23, 310–320 10.1007/s13361-011-0300-z 22161508

[64] Guan X., Zhang L. and Wypych J. (2018) Direct mass spectrometric characterization of disulfide linkages. MAbs 10, 572–582 10.1080/19420862.2018.1442998 29469657 PMC5973703

[65] Guo Y. and Marletta M.A. (2019) Structural insight into H-NOX gas sensing and cognate signaling protein regulation. Chembiochem 20, 7–19 10.1002/cbic.201800478 30320963

[66] Erbil W.K., Price M.S., Wemmer D.E. and Marletta M.A. (2009) A structural basis for H-NOX signaling in *Shewanella oneidensis* by trapping a histidine kinase inhibitory conformation. Proc. Natl. Acad. Sci. U.S.A. 106, 19753–19760 10.1073/pnas.0911645106 19918063 PMC2785238

[67] Pellicena P., Karow D.S., Boon E.M., Marletta M.A. and Kuriyan J. (2004) Crystal structure of an oxygen-binding heme domain related to soluble guanylate cyclases. Proc. Natl. Acad. Sci. U.S.A. 101, 12854–9 10.1073/pnas.0405188101 15326296 PMC516465

[68] Hespen C.W., Bruegger J.J., Phillips-Piro C.M. and Marletta M.A. (2016) Structural and functional evidence indicates selective oxygen signaling in *Caldanaerobacter subterraneus* H-NOX. ACS Chem. Biol. 11, 2337–2346 10.1021/acschembio.6b00431 27328180

[69] Olea C., Boon E.M., Pellicena P., Kuriyan J. and Marletta M.A. (2008) Probing the function of heme distortion in the H-NOX family. ACS Chem. Biol. 3, 703–710 10.1021/cb800185h 19032091 PMC2646007

[70] Olea C J., Herzik MA J., Kuriyan J. and Marletta M.A. (2010) Structural insights into the molecular mechanism of H-NOX activation. Protein Sci. 19, 881–887 10.1002/pro.357 20162612 PMC2867026

[71] Rao M., Herzik M.A., Iavarone A.T. and Marletta M.A. (2017) Nitric oxide-induced conformational changes govern H-NOX and histidine kinase interaction and regulation in *Shewanella oneidensis*. Biochemistry 56, 1274–1284 10.1021/acs.biochem.6b01133 28170222

[72] Guo Y., Iavarone A.T., Cooper M.M. and Marletta M.A. (2018) Mapping the H-NOX/HK binding interface in *Vibrio cholerae* by hydrogen/deuterium exchange mass spectrometry. Biochemistry 57, 1779–1789 10.1021/acs.biochem.8b00027 29457883

[73] Cremers C.M. and Jakob U. (2013) Oxidant sensing by reversible disulfide bond formation. J. Biol. Chem. 288, 26489–26496 10.1074/jbc.R113.462929 23861395 PMC3772196

[74] Bhattacharyya S., Ghosh S., Shant J., Ganguly N.K. and Majumdar S. (2004) Role of the W07-toxin on *Vibrio cholerae*-induced diarrhoea. Biochim. Biophys. Acta 1670, 69–80 10.1016/j.bbagen.2003.10.016 14729143

[75] Qadri F., Raqib R., Ahmed F., Rahman T., Wenneras C., Das S.K. et al. (2002) Increased levels of inflammatory mediators in children and adults infected with *Vibrio cholerae* O1 and O139. Clin. Diagn. Lab. Immunol. 9, 221–229 10.1128/cdli.9.2.221-229.2002 11874856 PMC119937

[76] Ellis C.N., LaRocque R.C., Uddin T., Krastins B., Mayo-Smith L.M., Sarracino D. et al. (2015) Comparative proteomic analysis reveals activation of mucosal innate immune signaling pathways during cholera. Infect Immun. 83, 1089–1103 10.1128/IAI.02765-14 25561705 PMC4333457

[77] Xu Q., Dziejman M. and Mekalanos J.J. (2003) Determination of the transcriptome of *Vibrio cholerae* during intraintestinal growth and midexponential phase in vitro. Proc. Natl. Acad. Sci. U.S.A. 100, 1286–1291 10.1073/pnas.0337479100 12552086 PMC298765

[78] Fong J.C., Syed K.A., Klose K.E. and Yildiz F.H. (2010) Role of Vibrio polysaccharide (vps) genes in VPS production, biofilm formation and *Vibrio cholerae* pathogenesis. Microbiology 156(Pt 9), 2757–2769 10.1099/mic.0.040196-0 20466768 PMC3068689

[79] Blow N.S., Salomon R.N., Garrity K., Reveillaud I., Kopin A., Jackson F.R. et al. (2005) *Vibrio cholerae* infection of Drosophila melanogaster mimics the human disease cholera. PLoS Pathog. 1, e8 10.1371/journal.ppat.0010008 16201020 PMC1238743

[80] Purdy A.E. and Watnick P.I. (2011) Spatially selective colonization of the arthropod intestine through activation of *Vibrio cholerae* biofilm formation. Proc. Natl. Acad. Sci. U.S.A. 108, 19737–19742 10.1073/pnas.1111530108 22106284 PMC3241763

[81] Faruque S.M., Islam M.J., Ahmad Q.S., Faruque A.S., Sack D.A., Nair G.B. et al. (2005) Self-limiting nature of seasonal cholera epidemics: Role of host-mediated amplification of phage. Proc. Natl. Acad. Sci. U.S.A. 102, 6119–6124 10.1073/pnas.0502069102 15829587 PMC1087956

[82] Faruque S.M., Biswas K., Udden S.M., Ahmad Q.S., Sack D.A., Nair G.B. et al. (2006) Transmissibility of cholera: in vivo-formed biofilms and their relationship to infectivity and persistence in the environment. Proc. Natl. Acad. Sci. U.S.A. 103, 6350–6355 10.1073/pnas.0601277103 16601099 PMC1458881

[83] Oh E., Kim J.C. and Jeon B. (2016) Stimulation of biofilm formation by oxidative stress in *Campylobacter jejuni* under aerobic conditions. Virulence 7, 846–851 10.1080/21505594.2016.1197471 27268722 PMC5029310

