# Supplementary material for: Structural insights into the mechanism of oxidative activation of heme-free H-NOX from Vibrio cholerae
Source: Biochem J. 2020 Mar 23;477(6):1123–36. doi: 10.1042/BCJ20200124 (PMC7108781; doi:10.1042/BCJ20200124)
Supplement: Supplementary Tables S1-S10 and Figures S1-S5 [file BCJ-477-1123-s1.pdf]

Supporting Information for:
Structural Insights into the Mechanism of Oxidative Activation of Heme-Free H-NOX from *Vibrio cholerae*

Roma Mukhopadhyay[a], Kelly Chacón[b], Jacqueline M. Jarvis[c], Marat Talipov[a], and Erik T. Yukl[a,1]

[a]Department of Chemistry and Biochemistry, New Mexico State University, Las Cruces, NM 88003.
[b]Department of Chemistry, Reed College, Portland, OR 97202.
[c]Department of Plant and Environmental Sciences, New Mexico State University, Las Cruces, NM 88003.
Running title: H-NOX Redox Sensing

Table S1. Additional details for the molecular dynamics simulations.

**Oxidation state of *Vc* HNOX**	**Model Name**	**Residue information**	**Composition of the periodic cell**	**Total Simulation Time, μs**
<u>R</u>educed	**R1**	Cysteine side chains deprotonated, Histidine side chains deprotonated at Nδ and protonated at Nε	2850 protein atoms, 12 Na ions, 1 Zn dication; total 8482 atoms	23.4
	**R2**	Cysteine side chains deprotonated, Histidine side chains deprotonated at Nδ and protonated at Nε<br>*MM/GBSA was not performed for R2' due to its kinetic instability	2852 protein atoms, 11 Na ions, 1 Zn dication; total 8672 atoms	3.9
<u>P</u>artially oxidized	**P1**	Histidine side chains deprotonated at Nδ and protonated at Nε. Cys174 and Cys182 side chains deprotonated, Cys149-Cys177 are covalently connected by a disulfide bridge.	2852 protein atoms, 9 Na ions, 1 Zn dication; total 8485 atoms	1.3
	**P1'**	Histidine side chains deprotonated at Nδ and protonated at Nε. Cys149 and Cys177 side chains deprotonated, Cys174-Cys182 are covalently connected by a disulfide bridge.	2852 protein atoms, 9 Na ions, 1 Zn dication; total 8485 atoms	1.3
	**P2**	Histidine side chains deprotonated at Nδ and protonated at Nε. Cys149 and Cys182 side chains deprotonated, Cys174-Cys177 are covalently connected by a disulfide bridge.	2852 protein atoms, 9 Na ions, 1 Zn dication; total 8485 atoms	1.3
	**P2'**	Histidine side chains deprotonated at Nδ and protonated at Nε. Cys174 and Cys177 side chains deprotonated,	2852 protein atoms, 9 Na ions, 1 Zn dication; total 8485 atoms	1.3

		Cys149-Cys182 are covalently connected by a disulfide bridge.		
Fully **O**xidized	**O1**	Histidine side chains deprotonated at Nδ and protonated at Nε. Cys149-Cys177 and Cys174-Cys182, form two disulfide bridges, respectively.	2852 protein atoms, 7 Na ions, 1 Zn dication; total 8485 atoms	1.3
	**O2**	Histidine side chains deprotonated at Nδ and protonated at Nε. Cys174-Cys177 and Cys149-Cys182, form two disulfide bridges, respectively.	2852 protein atoms, 9 Na ions, 7 Zn dication; total 8938 atoms	1.3

Table S2. Summary of the Umbrella Sampling simulations along the distance between the sulfur atom of Cys149 and Zn dication. The Table shows the restrain distance for the harmonic potential (R_{harm}), as well as the average and standard deviation values for this distance from the restrained MD simulations.

R_{harm}, Å	R_{mean}, Å	S.D., Å
1.7	1.92	0.03
1.8	1.94	0.03
2.0	1.97	0.04
2.2	2.02	0.04
2.4	2.09	0.05
2.5	2.15	0.07
2.6	2.23	0.09
2.8	2.73	0.09
2.9	2.73	0.18
3.0	3.06	0.08
3.1	3.09	0.09
3.2	3.26	0.08
3.4	3.46	0.08
3.6	3.66	0.07
3.8	3.83	0.07
4.0	3.99	0.07
4.2	4.17	0.07
4.4	4.33	0.07
4.6	4.51	0.08
4.8	4.74	0.08

5.0	4.94	0.08
5.2	5.16	0.08
5.4	5.38	0.08
5.6	5.59	0.08
5.8	5.79	0.08
6.0	5.99	0.08
6.2	6.18	0.08
6.4	6.39	0.08
6.6	6.59	0.08
6.8	6.77	0.08
7.0	6.98	0.08

Table S3. Summary of the Umbrella Sampling simulations along the distance between the sulfur atom of Cys174 and Zn dication. The Table shows the restrain distance for the harmonic potential (R_{harm}), as well as the average and standard deviation values for this distance from the restrained MD simulations.

R_{harm}, Å	R_{mean}, Å	S.D., Å
1.7	1.92	0.03
1.8	1.93	0.03
2.0	1.97	0.03
2.2	2.01	0.04
2.4	2.10	0.05
2.5	2.12	0.05
2.6	2.22	0.07
2.7	2.48	0.18
2.8	2.76	0.08
3.0	3.02	0.08
3.2	3.26	0.09
3.4	3.47	0.08
3.6	3.67	0.08
3.8	3.85	0.08
4.0	4.03	0.07
4.2	4.19	0.07
4.4	4.37	0.07
4.6	4.58	0.07
4.8	4.76	0.07
5.0	4.98	0.08
5.2	5.20	0.09

5.4	5.42	0.08
5.6	5.62	0.08
5.8	5.82	0.08
6.0	6.02	0.07
6.2	6.21	0.08
6.4	6.40	0.07
6.6	6.60	0.08
6.8	6.80	0.08
7.0	6.99	0.08

Table S4. Summary of the Umbrella Sampling simulations along the distance between the sulfur atom of Cys177 and Zn dication. The Table shows the restrain distance for the harmonic potential (R_{harm}), as well as the average and standard deviation values for this distance from the restrained MD simulations.

R_{harm}, Å	R_{mean}, Å	S.D., Å
1.8	1.93	0.03
2.0	1.97	0.03
2.2	2.02	0.04
2.4	2.08	0.05
2.5	2.54	0.08
2.5	2.13	0.06
2.6	2.64	0.08
2.8	2.92	0.08
2.8	2.90	0.08
3.0	3.11	0.07
3.2	3.29	0.08
3.4	3.49	0.07
3.6	3.70	0.08
3.8	3.89	0.08
4.0	4.08	0.08
4.2	4.30	0.08
4.4	4.48	0.08
4.6	4.70	0.08
4.8	4.86	0.08
5.0	5.13	0.09
5.2	5.37	0.07
5.4	5.56	0.08
5.6	5.73	0.07

5.8	5.93	0.07
6.0	6.11	0.08
6.2	6.27	0.07
6.4	6.48	0.07
6.6	6.64	0.07
6.8	6.82	0.07
7.0	7.00	0.07

Table S5. Summary of the Umbrella Sampling simulations along the distance between the sulfur atom of Cys182 and Zn dication. The Table shows the restrain distance for the harmonic potential (R_{harm}), as well as the average and standard deviation values for this distance from the restrained MD simulations.

R_{harm}, Å	R_{mean}, Å	S.D., Å
1.7	1.91	0.03
1.8	1.92	0.03
2.0	1.95	0.03
2.2	1.99	0.04
2.4	2.09	0.05
2.6	2.39	0.13
2.8	2.72	0.09
3.0	2.94	0.08
3.2	3.15	0.08
3.4	3.41	0.08
3.6	3.59	0.08
3.8	3.81	0.07
4.0	4.00	0.08
4.2	4.20	0.07
4.4	4.37	0.08
4.6	4.56	0.07
4.8	4.79	0.08
5.0	4.93	0.08
5.2	5.15	0.08
5.4	5.41	0.07
5.6	5.61	0.08
5.8	5.84	0.08
6.0	6.02	0.08
6.2	6.23	0.08
6.4	6.38	0.08

6.6	6.61	0.07
6.8	6.80	0.08
7.0	6.99	0.08

Table S6. Summary of the Umbrella Sampling simulations along the distance between the Nδ atom of His176 and Zn dication. The Table shows the restrain distance for the harmonic potential (R_{harm}), as well as the average and standard deviation values for this distance from the restrained MD simulations.

R_{harm}, Å	R_{mean}, Å	S.D., Å
1.7	1.92	0.04
1.8	1.94	0.04
2.0	2.02	0.05
2.2	2.14	0.06
2.3	2.41	0.08
2.4	2.56	0.08
2.6	2.75	0.08
2.8	2.88	0.08
2.9	2.95	0.08
3.0	3.16	0.07
3.2	3.33	0.07
3.4	3.51	0.07
3.6	3.68	0.07
3.8	3.87	0.08
4.0	4.05	0.08
4.2	4.24	0.08
4.4	4.42	0.08
4.6	4.63	0.08
4.8	4.83	0.08
5.0	5.03	0.08
5.2	5.22	0.08
5.4	5.40	0.08
5.6	5.60	0.08
5.8	5.79	0.08
6.0	6.03	0.08
6.2	6.22	0.08
6.4	6.42	0.07
6.6	6.62	0.08
6.8	6.80	0.08

7.0	6.98	0.08

Table S7. Summary of the Umbrella Sampling simulations along the distance between the Nδ atom of His181 and Zn dication. The Table shows the restrain distance for the harmonic potential (R_{harm}), as well as the average and standard deviation values for this distance from the restrained MD simulations.

R_{harm}, Å	R_{mean}, Å	S.D., Å
1.700	2.010	0.05
1.825	2.058	0.05
1.950	2.130	0.06
2.000	2.163	0.06
2.125	2.245	0.07
2.250	2.355	0.07
2.375	2.470	0.08
2.500	2.591	0.08
2.625	2.717	0.08
2.750	2.867	0.08
2.875	2.974	0.08
3.000	3.112	0.08
3.125	3.221	0.08
3.250	3.376	0.07
3.375	3.470	0.07
3.500	3.598	0.08
3.625	3.732	0.07
3.750	3.851	0.07
3.875	3.949	0.07
4.000	4.063	0.07
4.125	4.170	0.07
4.250	4.293	0.07
4.375	4.413	0.07
4.500	4.543	0.07
4.625	4.647	0.08
4.750	4.760	0.07
4.875	4.891	0.08
5.000	5.020	0.075
5.125	5.100	0.07
5.250	5.244	0.08
5.375	5.360	0.08
5.500	5.632	0.075

5.625	5.739	0.074
5.750	5.848	0.073
5.875	5.966	0.073
6.000	6.084	0.074
6.125	6.221	0.077
6.250	6.322	0.075
6.375	6.457	0.076
6.500	6.564	0.075
6.625	6.774	0.075
6.750	6.831	0.075
6.875	6.919	0.072
7.000	7.064	0.075

Table S8. Summary of the Umbrella Sampling simulations along the distance between the Gln171 group and Zn dication. The Table shows the restrain distance for the harmonic potential (R_{harm}), as well as the average and standard deviation values for this distance from the restrained MD simulations.

R_{harm}, Å	R_{mean}, Å	S.D., Å
1.700	1.797	0.037
1.800	1.823	0.040
2.200	1.959	0.057
2.400	2.132	0.073
2.600	2.350	0.085
2.700	2.677	0.101
2.800	2.858	0.073
2.000	1.885	0.048
3.200	3.253	0.076
3.400	3.485	0.074
3.600	3.657	0.071
3.800	3.839	0.072
3.000	3.073	0.077
4.200	4.162	0.073
4.400	4.338	0.076
4.600	4.547	0.084
4.800	4.772	0.085
4.000	3.988	0.077
5.200	5.219	0.080
5.400	5.428	0.079

5.600	5.625	0.080
5.800	5.837	0.079
5.000	4.994	0.082
6.200	6.230	0.077
6.400	6.423	0.076
6.600	6.601	0.075
6.800	6.791	0.078
6.000	6.034	0.081
7.200	7.168	0.076
7.400	7.352	0.078
7.600	7.546	0.076
7.800	7.763	0.072
7.000	6.997	0.074
8.200	8.123	0.074
8.400	8.329	0.078
8.600	8.598	0.082
8.800	8.803	0.077
8.000	7.942	0.079
9.000	8.980	0.077

Table S9. Predicted and observed m/z for monoisotopic masses of y- and b-ions from MS^3 (HCD) spectra of Gln147-Lys161 (m/z = 1429.87 (+1)) (Figure 5B).

**Ion**	**Pred. m/z (charge)**	**Obs. m/z (charge)**
b_2	242.15 (+1)	242.15 (+1)
y_3	289.15 (+1)	289.31 (+1)
y_4	346.21 (+1)	346.15 (+1)
b_5	487.23 (+1)	487.30 (+1)
y_5	459.29 (+1)	459.30 (+1)
y_6	572.38 (+1)	572.35 (+1)
y_7	685.46 (+1)	685.45 (+1)
y_8	742.48 (+1)	742.57 (+1)
b_9	857.42 (+1)	857.42 (+1)
y_9	871.52 (+1)	871.42 (+1)
b_{10}	970.50 (+1)	970.54 (+1)
y_{10}	942.56 (+1)	942.76 (+1)
y_{11}	1013.60 (+1)	1013.65 (+1)
b_{12}	1140.61 (+1)	1140.59 (+1)
y_{12}	1084.64 (+1)	1084.73 (+1)

Table S10. Predicted and observed m/z for monoisotopic masses of MS^3 (HCD) spectra of Ile169-Ser191 (m/z = 1263.13 (+2)) (Fig. 5C). Predicted masses assume a disulfide bond between Cys174 and Cys181 except where indicated by *, which assumes no disulfide bond (all Cys side chains are protonated).

Ion	Pred. m/z (charge)	Obs. m/z (charge)
b_3	343.20 (+1)	343.30 (+1)
y_3	316.19 (+1)	316.21 (+1)
b_5	539.32 (+1)	539.34 (+1)
*b_{13}	*1393.58 (+1)	1394.50 (+1)
b_{14}	1494.59 (+1)<br>747.80 (+2)	1493.60 (+1)<br>747.39 (+2)
b_{15}	1623.63 (+1)<br>812.31 (+2)	1623.76 (+1)<br>812.33 (+2)
b_{16}	1736.72 (+1)<br>868.86 (+2)	1736.93 (+1)<br>868.94 (+2)
b_{17}	1835.79 (+1)<br>918.40 (+2)	1835.74 (+1)<br>918.45 (+2)
b_{18}	1934.85 (+1)<br>967.93 (+2)	1934.80 (+1)<br>967.95 (+2)
b_{19}	1032.45 (+2)	1032.49 (+2)
b_{20}	1105.98 (+2)	1106.01 (+2)
b_{21}	1162.53 (+2)	1162.56 (+2)

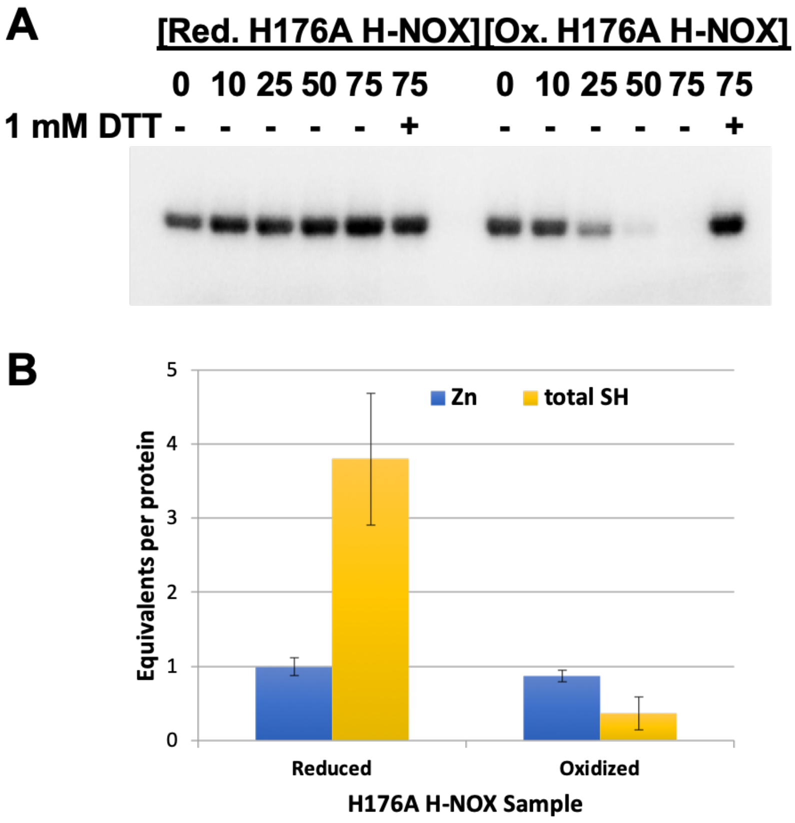

Figure S1. (A) Autophosphorylation of HnoK in the presence of increasing concentrations of heme-free H176A *Vc* H-NOX in the reduced (left) and oxidized (right) forms. 1 mM DTT was added to one sample to demonstrate reversibility. (B) Quantitation of zinc (blue bars) and total thiol (yellow bars) for heme-free H176A *Vc* H-NOX in the reduced (left) and oxidized (right) forms.

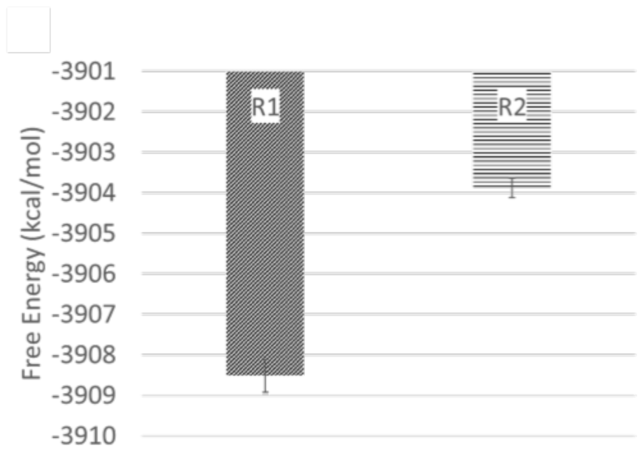

Figure S2. Relative free energy based on MM/GBSA calculation for reduced H-NOX models **R1** and **R2.**

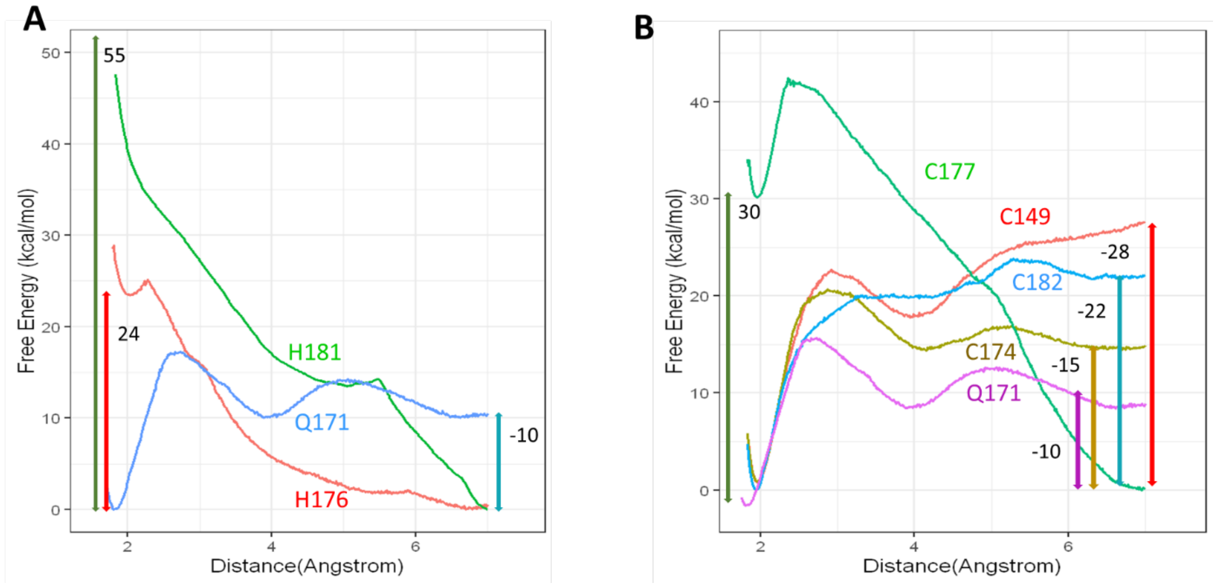

Figure S3. Potential Mean Force (PMF) plots generated from Umbrella sampling results for the different residues near the zinc site in reduced H-NOX (A) H176, H181 and Q171, and (B) C149, C174, C182, Q171 and C177.

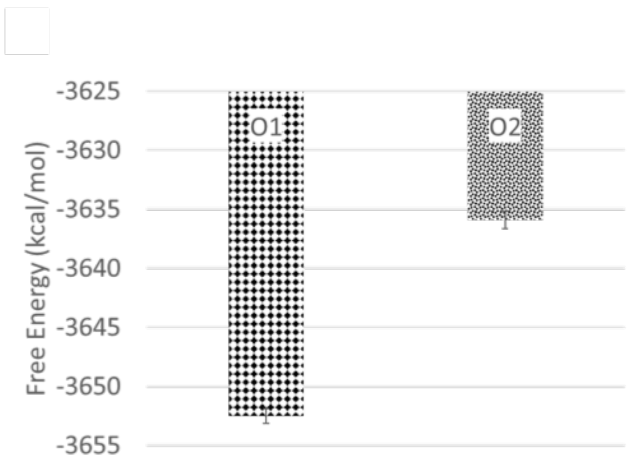

Figure S4. Relative free energy based on MM/GBSA calculation for full oxidized H-NOX models **O1** and **O2.**

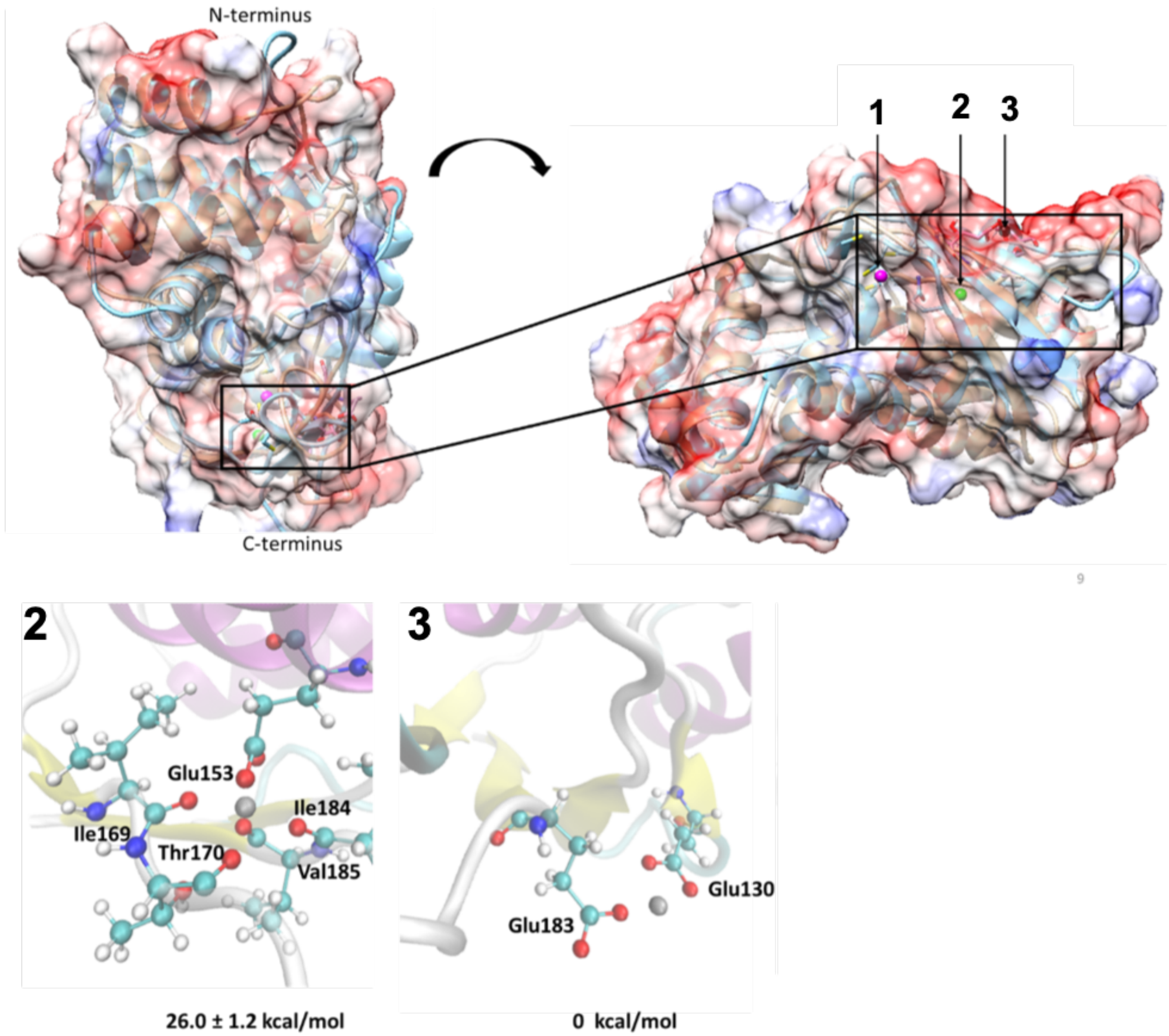

Figure S5. Electrostatic potential surface map for H-NOX protein was produced using Chimera software by selecting the Coulombic surface coloring method. The red region corresponds to the negatively charged surface, the white region to the neutral and the blue to the positively charged surface. The mauve sphere represents zinc in reduced H-NOX (1, see Figure 2), the green sphere for zinc in oxidized H-NOX after initial cMD simulation (2) and the black sphere for zinc in the most stable oxidized model (3). Molecular graphics and analysis was performed with UCSF Chimera, developed by the Resource for Biocomputing, Visualization, and Informatics at the University of California, San Francisco, with support from NIH P41-GM103311.
